# Evaluation of Phenolic Profile and Antioxidant Activity of Eleven Pistachio Cultivars (*Pistacia vera* L.) Cultivated in Andalusia

**DOI:** 10.3390/antiox11040609

**Published:** 2022-03-22

**Authors:** José Manuel Moreno-Rojas, Isabel Velasco-Ruiz, María Lovera, José Luis Ordoñez-Díaz, Víctor Ortiz-Somovilla, Elsy De Santiago, Octavio Arquero, Gema Pereira-Caro

**Affiliations:** 1 Department of Agroindustry and Food Quality Area, Andalusian Institute of Agricultural and Fisheries Research and Training (IFAPA), Alameda del Obispo, Avda. Menéndez-Pidal, s/n, 14004 Córdoba, Spain; t72verum@uco.es (I.V.-R.); josel.ordonez@juntadeandalucia.es (J.L.O.-D.); victor.ortiz@juntadeandalucia.es (V.O.-S.); mariag.pereira@juntadeandalucia.es (G.P.-C.); 2 Department of Agri-Food Engineering and Technology, Andalusian Institute of Agricultural and Fisheries Research and Training (IFAPA), Alameda del Obispo, Avda. Menéndez-Pidal, s/n, 14004 Córdoba, Spain; maria.lovera@juntadeandalucia.es (M.L.); octavio.arquero@juntadeandalucia.es (O.A.)

**Keywords:** pistachios, kernels, polyphenols, antioxidant activity, mass spectrometry, *Pistacia vera* L.

## Abstract

Pistachio (*Pistacia vera* L.) is a nut with a good adaptability to the Mediterranean conditions of cultivation, specifically in the Andalusian region, becoming an emerging crop. Moreover, it has been getting attention in the past years for the great content of bioactive compounds such as polyphenols. Although some studies have reported the polyphenolic profile of pistachios, most of them have analyzed the hull part, considered as a residue, and not the kernel which is the edible part. Therefore, characterization of eleven varieties of pistachios kernels cultivated in Andalusia and harvested in 2019 and 2020 was carried out by UHPLC-MS (ultra-high-performance liquid chromatography high-resolution mass spectrometry). The identification and quantification of 56 polyphenolic compounds was performed, being the hydroxybenzoic acids group the most abundant with a 71–86% of the total amount followed by flavan-3-ols group that accounted for 8–24%. Moreover, 3,4-dihydroxybenzoic acid was the main compound in most of the varieties, followed by vanillic acid hexoside. Larnaka, Avdat, Aegina, and Mateur presented the highest amount of total polyphenols, while Kalehghouchi, Joley, Lost Hills, Kerman, and Golden Hills were the varieties with the lowest content. Regarding the harvest season, no significant differences (*p* < 0.01) were found in the total amount of polyphenols between 2019 and 2020. In addition, the antioxidant activity was measured by DPPH (1,1-diphenyl-2-picryl-hydrazyl), ABTS (2,2′-azino-bis(3-ethylbenzothiazoline-6-sulfonic acid)), and ORAC (oxygen radical absorbance capacity) assays, showing a similar trend as that of the polyphenols.

## 1. Introduction

The pistachio (*Pistacia vera* L.) is a nut widely consumed throughout the world, originated from arid regions of Central and West Asia. This crop belongs to the *Anacardiaceae* family, and its health benefits and nutritional characteristics have been recently recognized by some studies [1,2].

The pistachio kernel or edible seed is commonly consumed as a natural or roasted salted snack food. Moreover, pistachio kernels are used in the industry as a food ingredient to elaborate pastry, ice cream, and confectionary production [1]. The edible kernel of pistachio has a thin layer which is a purplish skin covered by a hard shell, which is also covered by another shell known as hull (exocarp) characterized by turning the color from green to yellow-red depending on the ripeness [3].

According to the Food and Agriculture Organization (FAO), the total world pistachio production in 2020 was estimated at 1,125,305 tons [4] whereas around 42% was produced in the USA, followed by Turkey (26%), Iran (17%), China (7%), and Syria (6%). In the past, 62% of consumed pistachio in Spain was imported from Iran, increasing up to 91.5% in 2003 [5]. But during the last years, the Spanish pistachio production has been increasing due to an expansion of the regions where pistachios are grown [4]. The total Spanish production of pistachio in 2020 was estimated at 14,337 tons, being 16% produced in Andalusia [6]. In the same manner, the consumption per capita of pistachios has been growing in the last years. In Spain, the intake increased from 180 g per person per year in the 2019 to 220 g in 2020 [7].

Pistachio cultivation depends on several factors, hence not all parts of the country (Spain) are suitable for its production. It requires a minimum number of hours of cold weather which influence subsequent flowering and fertility. Likewise, the temperature, humidity, and soil characteristics of the Andalusian region favor the production and ripening of pistachio in a homogeneous way [8]. Hence, owing to the favorable climate, dry conditions, and moderately cold winters, pistachio is cultivated in the South of Spain, specifically in the region of Andalusia [9].

Pistachio nut is considered as a very important nutritional product and a good source of unsaturated fatty acids, of which 40–85% are MUFA and PUFA, being linoleic acid the most representative. It is also an important source of dietary fiber and protein. In addition, pistachio is rich in micronutrients such as magnesium, potassium, vitamin K, and tocopherol that contribute to a better glycemic index and the reduction of cardiovascular diseases [10,11,12].

Moreover, some studies have focused their attention on the analysis of the antioxidant activity and bioactive compounds present in pistachios, especially polyphenols, which are well-known for their ability to prevent the formation of pro-oxidants by blocking the action of reactive oxygen species and playing an important role in positive health effects such as cardioprotective, anti-diabetic, and anti-inflammatory effects [13,14]. Many of the studies published have investigated the polyphenolic profile from the hull part of pistachio [2,15,16,17,18,19,20], the purple skin of the kernel [21,22,23], and the hard shell covering the kernel [3].

Analyses related to the characterization of polyphenolic compounds in pistachio kernels are limited. Most research studied the antioxidant activity of the compounds found in the hull, and identified less compounds in the kernels compared to those found in the hull, and analyzed only one or a few varieties. According to the literature, the most commonly studied varieties of pistachio are Bronte (Italian variety), reporting mostly the presence of polyphenols such as quercetin and eriodictyol [24,25], as well as Kerman (described as a variety from America) with a higher amount of gallic acid and catechin [21,26]. In addition, Uzun and Ohadi (Turkeys varieties) have been studied by others authors [27,28], showing predominantly the presence of compounds such as chlorogenic acid and luteolin.

In the same way, several others publications were found in the literature with limited information. Arena et al. [29] reported a wider profile of polyphenols, but no information about the name of the varieties was mentioned, only the country of origin was indicated. Noguera-Artiaga et al. [30] published a study with a higher number of varieties such as Kerman, Avdat, Larnaka, Mateur, Napolitana, Aegina, Kastel, and Sirora, but reported only 13 polyphenolic compounds such as quercetin derivatives and kaempferol as the major ones. Likewise, other studies have evaluated the nutritional properties present in pistachios such as proteins, lipids, carbohydrates, minerals, as well as the fatty acid profile [12,31,32].

Therefore, due to the limited information found in literature regarding phenolic compounds of pistachio kernels, the aim of this study was to obtain an exhaustive polyphenolic profile by UHPLC-MS and antioxidant activity of eleven different varieties of pistachios cultivated in Andalusia (Spain) during two harvesting years (2019 and 2020).

## 2. Materials and Methods

### 2.1. Chemicals

Methanol (MeOH) HPLC grade, acetone, and potassium hydroxide were acquired from Panreac Applichem ITW Reagents (Darmstadt, Germany). Sodium chloride and magnesium chloride hexahydrate were purchased from Fisher Scientific (Madrid, Spain); sodium bicarbonate and ammonium carbonate were supplied by Sigma-Aldrich (Madrid, Spain); and potassium dihydrogen phosphate, sodium hydrogen carbonate, magnesium sulfate monohydrate, and potassium hydrogen phosphate were obtained from VWR International Eurolab (Barcelona, Spain). Reference standard compounds including gallic acid, pyrogallol (benzene-1,2-diol), ellagic acid, benzoic acid, chlorogenic acid, ferulic acid, sinapic acid, p-coumaric acid, luteolin, rutin, myricetin, quercetin, isorhamnetin, catechin, epigallocatechin gallate, and epicatechin gallate were purchased from Sigma-Aldrich (Madrid, Spain). The acetonitrile and methanol were of LC-MS grade.

### 2.2. Materials and Sample Preparation

Pistachios (*Pistacia vera* L.) were kindly provided from an experimental field of pistachio varieties of IFAPA in Guadix (Granada, Spain) which were planted in 2012. Eleven varieties were collected during two consecutive seasons (2019 and 2020): Aegina, Avdat, Golden Hills, Joley, Kalehghouchi, Kastel, Kerman, Larnaka, Lost Hills, Mateur and Sirora. The experimental design consisted in four blocks of random repetitions per variety. Fresh pistachios were peeled and ground using a homogenizer (SAMMIC, Madrid, Spain) and stored at −80 °C until analysis.

### 2.3. Extraction of Polyphenols

The extraction of polyphenols from pistachio samples was adapted from Ordóñez-Díaz et al. [33] with some modifications. One gram of sample was homogenized in an Ultra-Turrax (Stauten, Germany) with 12 mL of a methanol/acidified water mixture (80:20, *v/v*) with 0.1 % formic acid. The samples were centrifuged at 5000 rpm for 15 min at 4 °C, and supernatants were collected. The pellet was re-extracted following the same indications abovementioned. All the supernatants were pooled and made up to a final volume of 25 mL and stored at −80 °C until analysis.

### 2.4. Antioxidant Activity and Total Phenolic Content

#### 2.4.1. ABTS Assay

Free radical scavenging activity was measured using the ABTS (2,2′-azino-bis(3-ethylbenzothiazoline-6-sulfonic acid) decolorization method [34] with some modifications [35]). Briefly, the ABTS radical cation (ABTS^·+^) was produced by reacting ABTS^·+^ with a 2.45 mM of potassium persulfate solution stirring in dark at room temperature for 12–16 h before use. The ABTS^·+^ solution was diluted with ethanol to get a final absorbance of 0.8 ± 0.02 at 730 nm. Fresh ABTS^·+^ solution was prepared for each assay and day. Samples were resuspended in 500 µL of 7% methylated β-cyclodextrin (RMCD) in acetone/water (1:1, *v/v*) solution and 25 µL was added to 190 µL of ABTS^·+^ solution in a 96-well microplate measuring the absorbance every 20 s at 30 °C for over 6 min in a Synergy HTX Multi-Mode Microplate Reader (Biotek Instruments, Winooski, VT, USA). The antioxidant activity was expressed as mmol of Trolox equivalents per 100 g of sample (mmol TE/100 g). Each value is the average of three determinations.

#### 2.4.2. DPPH Assay

Free radical DPPH (1,1-diphenyl-2-picryl-hydrazyl) scavenging capacity was determined using the previously described methods by Sánchez-Moreno et al. [36]. The antioxidant activity was expressed as mmol of Trolox equivalents per 100 g of sample (mmol TE/100 g). Each value is the average of three measurements.

#### 2.4.3. ORAC Assay

Oxygen radical absorbance capacity (ORAC) assay was measured according to the method previously published by Huang et al. [37] and modified by Pereira-Caro et al. [35]. Briefly, samples were resuspended in 500 µL of 7% methylated β-cyclodextrin (RMCD) in acetone/water (1:1, *v/v*) solution. Then, 25 µL of either Trolox or sample extract or 75 mM phosphate buffer as a blank was added to a 96-well microplate followed by the addition of 150 µL of fluorescein work solution (8.5 × 10^−5^ mM) prepared in 75 mM phosphate buffer (pH 7.4). The microplate reader (Synergy HTX Multi-Mode Microplate Reader (Biotek Instruments, Winooski, VT, USA)) was programmed to record every two minutes for 120 min at 485 and 528 nm excitation and emission wavelengths, respectively, the fluorescence after the addition of 30 µL of AAPH (153 mM) as peroxyl radical generator, prepared in 75 mM phosphate buffer (pH 7.4). ORAC values are expressed as mmol Trolox equivalents per 100 g of sample (mmol TE/100 g).

#### 2.4.4. Total Phenolic Content

Total phenolic content (TPC) was determined by the Folin–Ciocalteu assay following the methodology of Slinkard and Singleton [38], with modifications of Hervalejo et al. [39] Total phenolic content was expressed as mmol of gallic acid per 100 g of sample (mmol GAE/100 g).

### 2.5. UHPLC-HRMS Polyphenol Analysis

Identification and quantification of polyphenols in the pistachio extracted samples were carried out by using UHPLC-HRMS mass spectrometer system (Thermo Scientific, San José, CA, USA) comprising a UHPLC pump, a PDA detector scanning from 200 to 600 nm, and an autosampler operating at 4 °C (ThermoFisher Scientific, San Jose, CA, USA).

Separation of phenolic compounds was performed on a Zorbax SB-C18 RRHD column (100 × 2.1 mm i.d., 1.8 µm (Agilent, Santa Clara, CA, USA) preceded by a guard pre-column of the same stationary phase and maintained at 40 °C. The flow rate was set to 0.2 mL/min with a 26 min gradient of phase A: deionized water with 0.1% formic acid and B: acetonitrile with 0.1% formic acid. The gradient started at 3% B, was maintained for 2 min, then rose to 65% B in 18 min, before rising to 80% B in 1 min and being maintained for 6 min with a 26 min gradient. After that, the column was equilibrated to the previous conditions within 10 min. After passing through the flow cell of the PDA detector part of the column, the eluate (0.2 mL/min) went into an Exactive Orbitrap mass spectrometer (Thermo Scientific, San José, CA, USA) fitted with a heated electrospray ionization probe (HESI) operating in negative ionization mode for the determination of polyphenols. Full scans were recorder in *m/z* range from 100 to 1200 with a resolution of 50,000 Hz and with a full AGC target of 100,000 charges, using 2 microscans. Analyses were also based on scans with in-source collision-induced dissociation (CID) at 25.0 eV. The capillary temperature of the MS experiment with HESI in negative ionization mode was at 320 °C, the sheath gas was 35 units, the heater temperature was 150 °C, the auxiliary gas was 10 units, and the spray voltage was 4.0 kV. Data acquisition and processing were carried out using Xcalibur 3.0 software (Thermo Scientific, San José, CA, USA).

Targeted identification of phenolic compounds was achieved comparing the exact mass and the retention time with available standards. In the absence of standards, compounds were tentatively identified by comparing the theoretical exact mass of the molecular ion with the measured accurate mass of the molecular ion and searched against metabolite databases including Metlin, Phenol Explorer and more general chemical databases such as PubChem and ChemSpider. Compounds having molecular masses within the pre-specified tolerance (≤10 ppm) of the query masses are retrieved from these databases. Quantification of phenolic compounds was carried out by selecting the theoretical exact mass of the molecular ion with reference to standard curves. In absence of reference compounds, they were quantified by reference to the calibration curve of a closely related parent compound.

### 2.6. Statistical Analysis

Two-way ANOVA and Tukey post-hoc tests were applied to identify the differences among samples using R software (v. 3.6.3, R Core Team, Vienna, Austria). A principal component analysis (PCA) was carried out for data exploration.

## 3. Results and Discussion

### 3.1. Antioxidant Activity

Total antioxidant activity was evaluated for the eleven varieties of pistachios following three scavenging methods: ABTS, DPPH, and ORAC assays. Results are displayed in Table 1 and significant statistical differences (*p* < 0.01) were observed among varieties. Larnaka, Mateur, Avdat, and Aegina were the varieties with the highest antioxidant capacity measured by ABTS assay. Regarding DPPH methodology, Larnaka presented the highest antioxidant activity, followed by Mateur, Avdat, and Aegina, while Golden Hills and Kalehghouchi varieties showing the lowest values. In the same way, Larnaka also presented higher values of antioxidant capacity by the ORAC assay, followed by Mateur, Avdat, and Aegina, while Joley, Kalehghouchi, and Golden Hills showed the lowest ones. The measurement of the total phenolic content (TPC) also showed Larnaka as the variety with the highest amount, followed by Aegina, Mateur, and Advat. In general, Larnaka, Mateur, Avdat, and Aegina showed the highest antioxidant activity values for the three scavenging methods and the total phenolic content. This finding agreed with Ojeda-Amador et al. [40] who reported Larnaka as the variety with the highest values for the antioxidant capacity evaluated by DPPH and ORAC assays followed by Aegina and Avdat. In the same manner, Noguera-Artiaga et al. [30] found similar results for Larnaka measured by ABTS radical but, in contrast with our findings in that study, Mateur variety had the lowest values. Significant differences (*p* < 0.01) in the antioxidant activity values measured by ABTS and DPPH, as well as in the TPC were found for the harvesting year, showing higher values for the samples harvested during 2020. This fact could be explained due to the different climatic conditions between years. No significant differences were shown for data measured by the ORAC method. To sum up, Larnaka variety presented the highest antioxidant activity in two of the three methods analyzed.

### 3.2. Polyphenols by UHPLC

A total of 56 polyphenolic compounds were identified and quantified by UHPLC-HRMS in the eleven varieties of pistachios from two seasons of harvest (2019 and 2020). Details of the characteristics for the identification of compounds, including the retention time (Rt), the experimental accurate mass, and the error (ppm) between the exact accurate mass and the mass found of the detected compounds are described in Table S1 (Supplementary Information). Polyphenolic compounds identified and quantified include thirteen hydroxybenzoic acids, ten galloyl derivatives, five hydroxycinnamic acids, four flavones, nine flavonols, nine flavan-3-ols, four flavanones, one flavanonol, and one stilbene.

Table 2 provides information about the total amount of the 56 polyphenolic compounds identified in the eleven varieties of pistachio grouped by families, for both 2019 and 2020 harvesting seasons. Regarding the qualitative profile among varieties, all of them showed a similar polyphenolic profile with the presence of all groups of polyphenols identified.

Significant differences (*p* < 0.01) among the eleven varieties were observed for the total content of polyphenols (Table 2). Larnaka, Avdat, Aegina, and Mateur presented the highest amount of the total polyphenols, which agrees with the antioxidant activity results. Based on that, we can conclude that the antioxidant activity is closely linked to the total polyphenols content. Most of the studies published on pistachios have characterized the hull part of the Turkey varieties [16,17,27,41,42] as well as the hulls of Bronte [18,21,22,26] and Kerman [2,43]. In agreement with our results, Noguera-Artiaga et al. [30] and Mannino et al. [22] have reported the Larnaka variety as one of the varieties with highest concentrations of polyphenolic compounds in pistachio kernels and seed skin respectively. However, in contrast, in the same study of Noguera-Artiaga et al. [30], found that Aegina and Avdat were the varieties with the lowest values for polyphenols. Furthermore, Kerman kernels has been widely studied and highly appreciated by the industry worldwide as a snack due to its quality [25,31,44].

In general, the group of the hydroxybenzoic acids was the most abundant for the eleven varieties under study, accounting for 71–86% of the total polyphenol content. Flavan-3-ols was the second more abundant group of polyphenols for all the varieties contributing from 8 to 24% of the total compounds. The rest of the seven groups of polyphenols were found as minority, representing less than 3% of the total content. 

Significant differences (*p* < 0.01) among varieties for each group of polyphenols, (V) were observed, except for the flavanonols. It could be highlighted that varieties with the lower values for the hydroxybenzoic acid group (Aegina Avdat, Kerman, Larnaka and Mateur) presented the highest values for the flavan-3-ols group, which may be due to the genetic synthesis mechanisms of polyphenols.

Regarding the harvesting season (S), in terms of the total polyphenols content (Table 2), no significant differences were found between the 2019 and 2020. Nevertheless, some significant differences were found for the individual polyphenol groups, but for the galloyl derivatives group. Those differences between years are compensated for the total amount of polyphenols. It was observed that the concentration of hydroxybenzoic and hydroxycinnamic acid groups, as well as flavanonols, tend to decrease significantly (*p* < 0.01) from 2019 to 2020, whereas for the rest of the groups increased. 

Considering that hydroxybenzoic acids group is the main contributor to the total polyphenols content, Table 3 shows detailed information for the thirteen identified and quantified compounds of this category in the eleven varieties of pistachios. Significant differences (*p* < 0.01) were observed for all the compounds for the varieties except for the benzoic acid derivative III. In this way, we can conclude that Larnaka, Avdat, Aegina, and Mateur presented three-fold concentration than the rest of varieties. The main compound was the 3,4-dihydroxybenzoic acid for most of the studied varieties, ranging from 43 to 67% of the total content of this group, followed by vanillic acid hexoside (28–51%) except for Kalehghouchi and Kerman for which the major one was the vanillic acid hexoside and 3,4-dihydroxybenzoic acid respectively. The rest of the compounds were found in minor amounts. Results are in agreement with the literature, where 3,4-dihydroxybenzoic acid was described as one of the principal compounds detected, for instance, in the shells of Bronte variety [45] and in unknown varieties from Turkey (name is not mentioned) [42]. In contrast with our results, Tomaino et al. [21], found quercetin-3-*O*-rutinoside as the most abundant polyphenol in the kernels from the pistachio studied variety (Bronte) while eriodictyol-7-*O*-glucoside and gallic acid were described as the main compounds in the skins. 

As mentioned before, the total amount of hydroxybenzoic acids decreased significantly (*p* < 0.01) between the two harvest years (2019 and 2020) because of 3,4-dihydroxybenzoic acid which is the major compound, since the tendency of most compounds is to increase. Nonetheless, no significant differences were found for theogallin, galloylshikimic acid, digallic acid, and benzoic acid derivative based on the harvesting year factor. 

Moreover, the nine compounds of the flavan-3-ols group (second more abundant group) are presented in Table 4, showing that Larnaka, Avdat, Aegina, and Mateur presented between six and eight-fold higher concentration than the rest of the varieties, for which no significant differences were observed for total of flavan-3-ols content. Epigallocatechin gallate was the most abundant compound (ranging from 39 to 83% of the total of this group), followed by catechin (7–22%), and gallocatechin (5–8%). Proanthocyanidins have been previously reported in pistachios hulls [18,43], in the skin of the seed [22,23], and in the oil obtained from pistachios [46]. Regarding pistachio kernels, Liu et al. [25] and Tomaino et al. [21] identified and quantified catechin and epicatechin by HPLC-DAD in Bronte and Kerman varieties. However, to our best knowledge, epiafzelechin 3-gallate, epicatechin gallate, and three afzelechin derivatives have been also detected for the first time in pistachio kernels.

In contrast with the hydroxybenzoic group, a significant (*p* < 0.01) increase was observed between 2019 and 2020 in flavan-3-ols. The trend in most of the compounds was the same, but for Afzelechin I, for which no significant differences were found. 

Moreover, additional information about the rest of the groups of polyphenols found as minority and representing less than 3% of the total content can be further checked in the supplementary material (Tables S3–S8). Due to the complexity of the univariate analysis and in order to facilitate the discussion and comprehension of the results, a multivariate approach is performed and discussed in the following section.

### 3.3. Multivariate Analysis of Data (PCA)

**Figure 1:**
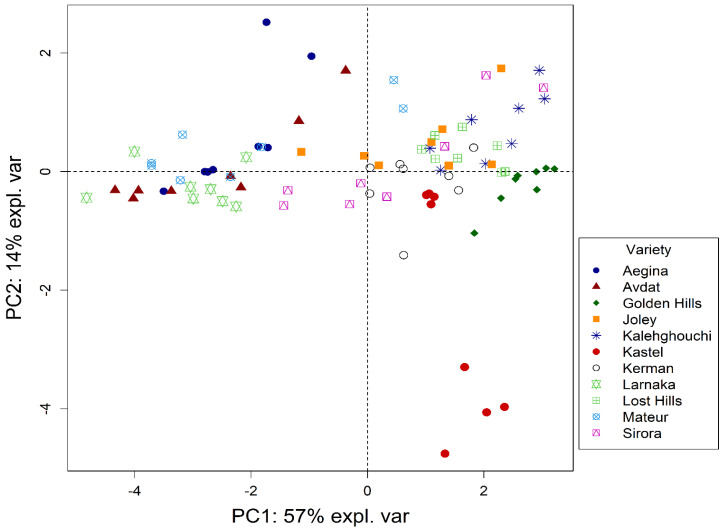
Scores plot of the principal component analysis (PCA) carried out on the eleven varieties of pistachios using the groups of polyphenols and antioxidant activity.

To determine the main variation sources in the data from the groups of polyphenols in pistachios, a PCA was constructed, and the subspace spanned by the first two principal components (PCs), which explained the 71% of the total variance, was plotted (Figure 1).

**Figure 2:**
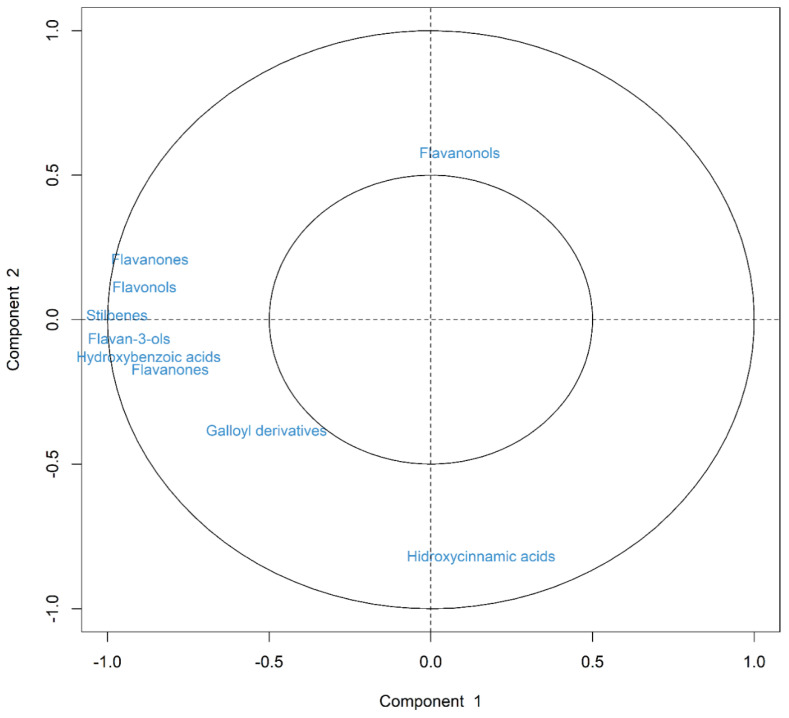
Loadings plot of the principal component analysis (PCA) carried out on the eleven varieties of pistachios using the groups of polyphenols and antioxidant activity.

Different clusters related to the different families of polyphenols in pistachios were observed. In the direction of PC1, which explained around 57% of the total variance, Larnaka, Avdat, Aegina, and Mateur varieties which are the most abundant in polyphenols were clearly separated from the ones with less content of compounds (Joley, Lost Hills, Kerman, Golden Hills, and Kalehghouchi). The main families of polyphenols involved in such differences were hydroxybenzoic acids, flavan3-ols, stilbenes, flavonols, and flavanones which could be primarily responsible for the high antioxidant activity measured by ABTS, DPPH, and ORAC assays, being present in the same square (Figure 2). Additionally, the PC2 explained the 14% of the total variance, showing the difference between Kastel and the rest of the varieties (Figure 2, bottom part), mainly due to the contribution of hydroxycinnamic acids (Table 2). Kastel variety presented the highest content of hydroxycinnamic acids on account of the great amount found in ferulic acid derivative (Table S3).

**Figure 3:**
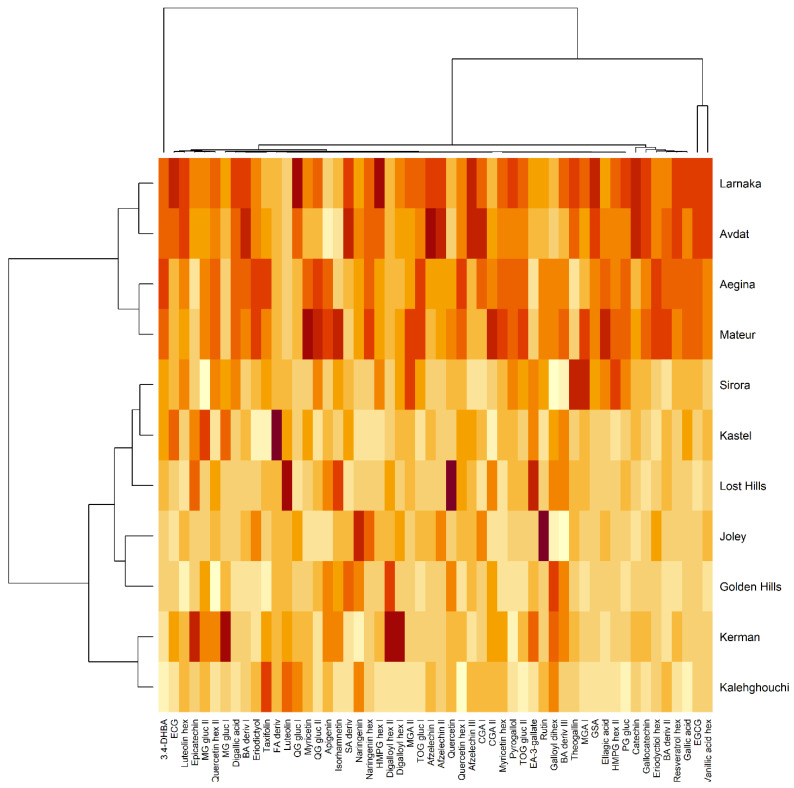
Hierarchical cluster analysis (HCA) carried out on the eleven varieties of pistachios using the polyphenolic compounds. 3,4-DHB: 3,4-dihydroxybenzoic acid. ECG: epicatechin gallate. MG gluc: mono-galloyl-glucose. BA: benzoic acid. FA: ferulic acid. QG: quercetin galloyl. SA: sinapic acid. HMPG: hydroxy-methoxyphenylgalloyl. MGA: methylgallic acid. CGA: chlorogenic acid. TOG: tetra-O-galloyl. EA: epiafzelechin. GSA: galloylshikimic acid. PG: pentagalloylglucose. EGCG: epigallocatechin gallate. Hex: hexoside. Gluc: glucoside. Deriv: derivative.

Moreover, for more detailed information related to the distribution of the 56 polyphenols, a hierarchical cluster analysis (HCA) was performed (Figure 3). This hierarchical chemotype clustering information reflects the classification of two groups of varieties, showing the cluster with highest concentrations of polyphenols found in Larnaka, Avdat, Aegina, and Mateur separated from the rest of the varieties with lower values. The analysis also emphasized that 3,4-dihydroxybenzoic acid (3,4-DHBA) is the major compound found in Larnaka, Avdat, Aegina, and Mateur varieties in agreement with the results previously discussed in Table 3. Likewise, another cluster is differentiated by vanillic acid hexoside and epigallocatechin gallate (EGCG), which follow the same trend, presenting the highest content in the four major varieties.

## 4. Conclusions

Results of this study report an exhaustive characterization of 56 polyphenolic compounds present in pistachio kernels from eleven varieties cultivated in Andalusia, describing hydroxybenzoic acids and flavan-3-ols as the most abundant groups. Likewise, the main polyphenolic compound was 3,4-dihydroxybenzoic acid for most of the varieties, followed by vanillic acid hexoside. Larnaka, Avdat, Aegina, and Mateur presented the highest amount of the total polyphenols, while Kalehghouchi, Joley, Lost Hills, Kerman, and Golden Hills were the varieties with the lowest content. Regarding the harvest season, no significant differences (*p* < 0.01) were found between 2019 and 2020 in the total amount of polyphenols. In addition, antioxidant activity was measured, showing Larnaka as the highest variety in two of the three methods analyzed without significant differences in the remaining varieties. Results suggest that antioxidant activity is mainly attributable to the content of the family of hydroxybenzoic acids. Moreover, the different results obtained among the antioxidant methods may be linked to the contribution of other antioxidant compounds in the hydrophobic fraction which react with the radicals. This study was carried out to characterize the polyphenols presented in the different varieties of pistachios and the variability of the compounds, since it is the basis for subsequent digestibility analyses, which are necessary to evaluate the bioaccessibility and bioavailability of these compounds.

## Figures and Tables

**Table 1:** Influence of variety (V) and season (S) on antioxidant capacity measured by ABTS, DPPH, ORAC, and TPC (mmol/100 g).

	ABTS	DPPH	ORAC	TPC
**Values ^a^**				
***Variety***				
Aegina	3.17 b	2.82 b	7.9 c	2.96 b
Avdat	3.20 ab	2.78 b	10.3 b	2.70 b
Golden Hills	1.28 f	0.86 e	3.6 fg	1.35 d
Joley	1.48 ef	1.09 de	3.5 g	1.57 cd
Kalehghouchi	1.55 def	0.93 e	3.5 g	1.45 d
Kastel	1.87 cd	1.22 cd	4.9 e	1.56 cd
Kerman	2.00 c	1.25 cd	5.3 e	1.91 c
Larnaka	3.53 a	3.22 a	15.8 a	3.51 a
Lost Hills	1.71 cde	1.22 cd	4.5 ef	1.63 cd
Mateur	3.33 ab	2.72 b	11.0 b	2.80 b
Sirora	1.97 c	1.48 c	6.4 d	1.31 d
***Season***				
2019	2.06 b	1.59 b	7.1	1.89 b
2020	2.51 a	1.97 a	6.9	2.25 a
**Significance ^b^**				
Variety (V)	***	***	***	***
Season (S)	***	***	ns	***
V × S	***	***	***	***

^a^ Average values. ^b^ Level of significance: * *p* ≤ 0.05, ** *p* ≤ 0.01, *** *p* ≤ 0.001, ns: non-significant. Values with different letters are significantly different as determined through Tukey test.

**Table 2:** Influence of the cultivar and harvesting season on the polyphenols profile groups (µg/100 g).

	Hydroxy Benzoic Acids	Galloyl Derivatives	Hydroxy Cinnamic Acids	Flavones	Flavonols	Flavan-3-ols	Flavanones	Flavanonols	Stilbenes	Total
**Values ^a^**										
***Variety***										
Aegina	380 a	5.0 abc	0.34 b	3.31 ab	4.31 a	105 bc	8.10 a	1.15	4.20 b	512 a
Avdat	406 a	5.0 abc	0.40 b	4.12 a	3.95 abcd	137 ab	6.24 b	0.90	5.22 a	569 a
Golden Hills	152 bcd	4.2 bcd	0.36 b	0.44 f	1.89 f	17 d	1.34 cd	0.44	1.40 d	179 bc
Joley	205 bcd	3.4 d	0.33 b	2.04 cde	3.14 de	29 d	5.22 b	0.68	2.12 cd	252 bc
Kalehghouchi	116 d	3.2 d	0.31 b	2.32 bcde	2.49 ef	17 d	1.60 cd	1.23	1.66 cd	146 c
Kastel	242 b	5.3 ab	1.87 a	1.96 de	2.49 ef	34 d	1.05 d	0.46	2.00 cd	290 b
Kerman	148 cd	6.2 a	0.31 b	2.03 cde	3.42 bcd	35 d	1.72 cd	0.83	2.47 c	200 bc
Larnaka	422 a	6.0 a	0.38 b	4.36 a	4.22 abc	144 a	6.04 b	0.76	4.92 ab	592 a
Lost Hills	199 bcd	3.6 cd	0.31 b	1.40 ef	3.15 de	19 d	1.70 cd	0.78	1.88 cd	231 bc
Mateur	366 a	5.3 ab	0.33 b	2.91 bcd	4.30 ab	101 c	8.31 a	0.95	4.08 b	494 a
Sirora	227 bc	4.3 bcd	0.33 b	3.18 abc	3.38 cd	49 d	2.42 c	0.88	2.41 c	292 b
***Season***										
2019	286 a	4.5	0.633 a	2.03 b	3.16 b	51 b	3.73 b	1.11 a	2.81 b	355
2020	235 b	4.9	0.326 b	3.08 a	3.52 a	74 a	4.22 a	0.54 b	3.08 a	328
**Significance ^b^**										
Variety (V)	***	***	***	***	***	***	***	ns	***	***
Season (S)	***	ns	***	***	**	***	**	***	*	ns
V × S	***	*	***	**	**	*	***	ns	*	**

^a^ Average values. ^b^ Level of significance: * *p* ≤ 0.05, ** *p* ≤ 0.01, *** *p* ≤ 0.001, ns: non-significant. Values with different letters are significantly different as determined through Tukey test.

**Table 3:** Influence of the cultivar and harvesting season on the hydroxybenzoic acid group (µg/100 g).

	Gallic Acid	Pyro- Gallol	Theogallin (3-Galloyl-Quinic Acid)	3, 4-DHB	GSA	Digallic Acid	MGA I	MGA II	Vanillic Acid Hexoside	Ellagic Acid	BA Deriv I	BA Deriv II	BA Deriv III	Total
**Values ^a^**														
***Variety***														
Aegina	4.98 a	0.66 ab	0.11 b	238 a	0.40 b	1.23 ab	0.32 cde	0.123 b	128 c	0.48 ab	1.16 bc	4.7 ab	0.40	380 a
Avdat	4.31 ab	0.62 ab	0.59 a	215 ab	0.56 a	1.30 ab	0.37 c	0.120 bc	176 a	0.46 ab	1.41 a	4.9 ab	0.36	406 a
Golden Hills	2.12 e	0.27 de	0.18 b	101 cde	0.13 c	0.23 c	0.16 ef	0.093 cd	44 e	0.21 b	0.62 f	2.4 d	0.40	152 bcd
Joley	2.16 e	0.31 de	0.22 b	111 cde	0.16 c	0.32 c	0.20 def	0.097 bcd	87 d	0.30 ab	0.89 de	2.8 cd	0.29	205 bcd
Kalehghouchi	1.28 e	0.28 de	0.19 b	50 e	0.10 c	0.14 c	0.13 f	0.082 d	59 de	0.13 b	0.68 f	3.4 bcd	0.35	116 d
Kastel	3.48b c	0.47 bcd	0.20 b	154 bc	0.16 c	0.36 c	0.34 cd	0.107 bcd	78 de	0.23 b	0.78 ef	3.3 bcd	0.41	242 b
Kerman	2.23 de	0.18 e	0.27 b	67 de	0.13 c	0.34 c	0.23 cdef	0.100 bcd	74 de	0.17 b	0.76 ef	2.1 d	0.40	148 cd
Larnaka	5.37 a	0.76 a	0.63 a	237 a	0.62 a	1.56 a	0.56 b	0.152 a	169 ab	0.39 ab	1.28 ab	4.4 abc	0.41	422 a
Lost Hills	2.32 de	0.40 cd	0.26 b	133 cd	0.17 c	0.34 c	0.29 cde	0.106 bcd	58 de	0.23 b	0.67 f	3.4 bcd	0.40	199 bcd
Mateur	4.94 a	0.70 a	0.11 b	217 ab	0.38 b	1.24 ab	0.67 ab	0.159 a	134 bc	0.61 a	1.07 cd	5.4 a	0.42	366 a
Sirora	3.25 cd	0.60 abc	0.78 a	153 bc	0.31 b	1.07 b	0.81 a	0.160 a	63 de	0.44 ab	0.69 f	2.2 d	0.31	227 bc
***Season***														
2019	4.07 a	0.29 b	0.31	189 a	0.29	0.78	0.26 b	0.100 b	87 b	0.53 a	0.91	2.1 b	0.35 b	286 a
2020	2.56 b	0.66 a	0.33	115 b	0.28	0.70	0.48 a	0.137 a	107 a	0.14b	0.91	5.0 a	0.40 a	235 b
**Significance ^b^**														
Variety (V)	***	***	***	***	***	***	***	***	***	***	***	***	ns	***
Season (S)	***	***	ns	***	ns	ns	***	***	***	***	ns	***	*	***
V × S	***	***	*	***	**	***	***	***	*	**	**	***	ns	***

^a^ Average values. ^b^ Level of significance: * *p* ≤ 0.05, ** *p* ≤ 0.01, *** *p* ≤ 0.001, ns: non-significant. Values with different letters are significantly different as determined through Tukey test. 3,4-DHB: 3,4-dihydroxybenzoic acid. GSA: galloylshikimic acid. MGA: methylgallic acid derivative. BA deriv: benzoic acid derivative.

**Table 4:** Influence of the cultivar and harvesting season on the flavan-3-ols group (µg/100 g).

	Gallo Catechin	Catechin	Epi Catechin	Epigallo Catechin Gallate	Epi Catechin Gallate	Epiafzelechin3-Gallate	Afzelechin I	Afzelechin II	Afzelechin III	Total
**Values ^a^**										
***Variety***										
Aegina	7.43 b	7.0 bc	0.98 e	86.8 ab	2.12 bc	0.30 e	0.110 b	0.1108 bc	0.16 bc	105 bc
Avdat	10.34 a	15.5 a	2.25 bcd	104.6 ab	3.60 a	0.40 cde	0.119 a	0.1198 a	0.23 a	137 ab
Golden Hills	1.13 d	3.3 de	1.41 cde	8.9 c	1.70 bcd	0.40 cde	0.105 d	0.1059 de	0.17 b	17 d
Joley	2.00 cd	2.8 e	1.16 de	21.2 c	1.37 cd	0.39 cde	0.107 bcd	0.1131 b	0.16 bc	29 d
Kalehghouchi	0.95 d	3.5 de	1.16 de	9.3 c	1.08 d	0.29 e	0.107 bcd	0.1058 e	0.17 b	17 d
Kastel	1.71 cd	5.8 bcde	2.46 bc	19.2 c	3.57 a	0.59 abc	0.107 bcd	0.1069 cde	0.17 b	34 d
Kerman	2.85 cd	6.9 bc	3.66 a	17.7 c	2.51 b	0.62 ab	0.106 cd	0.1049 e	0.13 d	35 d
Larnaka	10.43 a	16.0 a	2.51 bc	110.0 a	4.10 a	0.46 bcde	0.116 a	0.1190 a	0.25 a	144 a
Lost Hills	1.09 d	4.2 cde	3.08 ab	7.5 c	1.91 bc	0.78 a	0.106 cd	0.1061 de	0.16 bc	19 d
Mateur	8.14 ab	8.0 b	1.16 de	80.9 b	2.12 bc	0.32 de	0.108 bc	0.1100 bcd	0.17 b	101 c
Sirora	4.12 c	6.2 bcd	1.48 cde	33.9 c	2.14 bc	0.52 bcd	0.106 cd	0.1059 e	0.14 cd	49 d
***Season***										
2019	3.22 b	5.9 b	1.10 b	37.9 b	2.26 b	0.39 b	0.1086	0.1087 b	0.16 b	51 b
2020	5.90 a	8.5 a	2.77 a	53.0 a	2.50 a	0.53 a	0.1088	0.1109 a	0.18 a	74 a
**Significance ^b^**										
Variety (V)	***	***	***	***	***	***	***	***	***	***
Season (S)	***	***	***	***	*	***	ns	***	***	***
V × S	**	ns	**	**	ns	ns	*	*	***	*

^a^ Average values. ^b^ Level of significance: * *p* ≤ 0.05, ** *p* ≤ 0.01, *** *p* ≤ 0.001, ns: non-significant. Values with different letters are significantly different as determined through Tukey test.

## Data Availability

The data presented in this study are available in the article and supplementary materials.

## Supplementary Materials

The following supporting information can be downloaded at: https://www.mdpi.com/article/10.3390/antiox11040609/s1. Table S1: UHPLC-HRMS characteristics of the polyphenols identified in pistachios varieties. Table S2: Influence of the cultivar and harvesting season on the galloyl derivatives group. Table S3: Influence of the cultivar and harvesting season on the hydroxycinnamic acids group. Table S4: Influence of the cultivar and harvesting season on the flavones group. Table S5: Influence of the cultivar and harvesting season on the flavonols group. Table S6: Influence of the cultivar and harvesting season on the flavanones group. Table S7: Influence of the cultivar and harvesting season on the flavanonols group. Table S8: Influence of the cultivar and harvesting season on the stilbenes group.
