# Supplementary material for: Evaluation of Phenolic Profile and Antioxidant Activity of Eleven Pistachio Cultivars (Pistacia vera L.) Cultivated in Andalusia
Source: Antioxidants (Basel). 2022 Mar 22;11(4):609. doi: 10.3390/antiox11040609 (PMC9029509; doi:10.3390/antiox11040609)
Supplement: Supplementary file 1 [file antioxidants-11-00609-s001.zip › antioxidants-1632196-supplementary.pdf]

SUPPLEMENTARY INFORMATION:

Evaluation of phenolic profile and antioxidant activity of eleven pistachio cultivars (*Pistacia vera* L.) cultivated in Andalusia

José Manuel Moreno-Rojas[1]*, Isabel Velasco-Ruiz[1], María Lovera[2], José Luis Ordoñez-Díaz1, Víctor Ortíz-Somovilla[1], Elsy De Santiago[1]*, Octavio Arquero[2], Gema Pereira-Caro[1]

1 Department of Agroindustry and Food Quality Area, Andalusian Institute of Agricultural and Fisheries Research and Training (IFAPA), Alameda del Obispo, Avda. Menéndez-Pidal, s/n. 14004, Córdoba, Spain. elsyg.santiago@juntadeandalucia.es (E.DS.); t72verum@uco.es (I. V.-R); josel.ordonez@juntadeandalucia.es (J.L.O.-D.); mariag.pereira@juntadeandalucia.es (G.P.-C.); victor.ortiz.somovilla@juntadeandalucia.es (V.O.-S.),

2 Department of Agri-Food Engineering and Technology, Andalusian Institute of Agricultural and Fisheries Research and Training (IFAPA), Alameda del Obispo, Avda. Menéndez-Pidal, s/n. 14004, Córdoba, Spain. maria.lovera@juntadeandalucia.es (M.L.); octavio.arquero@juntadeandalucia.es (O.A).

* Correspondence: josem.moreno.rojas@juntadeandalucia.es

elsyg.santiago@juntadeandalucia.es

Table S1. UHPLC-HRMS Characteristics of the polyphenols identified among pistachios varieties

RT (min)	Compound	Chemical Formula	$[m/z]^-$ Theoretical	Error (ppm)	MSIMI level[a]
	Hydroxybenzoic acids				
3.27	Gallic acid	$C_7H_6O_5$	169.0131	-1.18	1
3.49	Pyrogallol (Benzene-1,2-diol)	$C_6H_5O_3$	125.0233	-0.80	1
3.8	Theogallin (3-Galloyl-quinic acid)	$C_{14}H_{16}O_{10}$	343.0663	-0.87	2
6.48	3,4-Dihydroxybenzoic acid	$C_7H_6O_4$	153.0182	-1.31	2
6.88	Galloylshikimic acid	$C_{14}H_{14}O_9$	325.0554	3.38	2
8.75	Digallic acid	$C_{14}H_{10}O_9$	321.0241	2.18	2
8.81	Methylgallic acid derivative I	$C_8H_7O_5$	183.0287	1.64	2
11.88	Methylgallic acid derivative II	$C_8H_7O_5$	183.0287	1.64	2
10.09	Vanillic acid hexoside	$C_{14}H_{18}O_9$	329.0867	3.34	2
11.08	Ellagic acid	$C_{14}H_6O_8$	300.9978	2.99	1
10.46	Benzoic acid derivative I	$C_8H_7O_4$	167.0338	2.39	1
11.97	Benzoic acid derivative II	$C_8H_7O_4$	167.0338	2.39	1
14.04	Benzoic acid derivative III	$C_8H_7O_4$	167.0338	2.39	1
	Galloyl derivatives				
2.67	Mono-galloyl-glucose I	$C_{13}H_{16}O_{10}$	331.0659	3.02	2
6.63	Mono-galloyl-glucose II	$C_{13}H_{16}O_{10}$	331.0659	3.02	2
3.6	Galloyl dihexose	$C_{19}H_{26}O_{15}$	493.1188	0.20	2
8.46	Digalloyl hexose I	$C_{20}H_{20}O_{14}$	483.0769	2.28	2
9.02	Digalloyl hexose II	$C_{20}H_{20}O_{14}$	483.0769	2.28	2
9.05	Hydroxy-methoxyphenylgalloyl hexoside I	$C_{20}H_{22}O_{12}$	453.1027	2.43	2
9.75	Hydroxy-methoxyphenylgalloyl hexoside II	$C_{20}H_{22}O_{12}$	453.1027	2.43	2
9.96	Tetra-O-galloyl glucoside I	$C_{34}H_{28}O_{19}$	787.0994	1.40	2
10.7	Tetra-O-galloyl glucoside II	$C_{34}H_{28}O_{19}$	787.0994	1.40	2
11.23	Pentagalloylglucose	$C_{41}H_{32}O_{26}$	939.1098	0.64	2
	Hydroxycinnamic acids				
8.19	Chlorogenic acid I	$C_{16}H_{18}O_9$	353.0867	0.28	1
9.17	Chlorogenic acid II	$C_{16}H_{18}O_9$	353.0867	0.28	1
8.59	Ferulic acid derivative	$C_{10}H_{10}O_4$	193.0516	1.55	1
9.02	Sinapic acid derivative	$C_{11}H_{12}O_5$	223.0600	2.24	1
10.83	*p*-Coumaric acid	$C_9H_8O_3$	163.0395	0.61	1

	Flavones				
8.65	Luteolin hexoside	$C_{21}H_{20}O_{11}$	447.0921	1.79	2
14.27	Luteolin	$C_{15}H_{10}O_6$	285.0393	3.16	1
11.14	Apigenin hexoside	$C_{21}H_{20}O_{10}$	431.0972	1.16	2
13.71	Apigenin	$C_{15}H_{10}O_5$	269.0444	3.35	2
	Flavonols				
10.87	Rutin	$C_{27}H_{30}O_{16}$	609.145	0.82	1
10.35	Myricetin hexoside	$C_{21}H_{20}O_{13}$	479.082	1.67	2
12.65	Myricetin	$C_{15}H_{10}O_8$	317.0291	1.89	1
10.69	Quercetin hexoside I	$C_{21}H_{20}O_{12}$	463.0871	1.73	2
11.15	Quercetin hexoside II	$C_{21}H_{20}O_{12}$	463.0871	1.73	2
10.76	Quercetin galloyl glucoside I	$C_{28}H_{24}O_{16}$	615.098	1.46	2
11.57	Quercetin galloyl glucoside II	$C_{28}H_{24}O_{16}$	615.098	1.46	2
14.32	Quercetin	$C_{15}H_{10}O_7$	301.0342	3.32	1
14.85	Isorhamnetin	$C_{16}H_{12}O_7$	315.0499	4.76	1
	Flavan-3-ols				
6.72	Gallocatechin	$C_{15}H_{14}O_7$	305.0655	3.61	2
8.81	Catechin	$C_{15}H_{14}O_6$	289.0707	2.77	1
9.69	Epicatechin	$C_{15}H_{14}O_6$	289.0707	2.42	2
9.82	Epigallocatechin gallate	$C_{22}H_{18}O_{11}$	457.0765	0.44	1
11.18	Epicatechin gallate	$C_{22}H_{18}O_{10}$	441.0816	2.27	1
12.05	Epiafzelechin 3-gallate	$C_{22}H_{18}O_9$	425.0867	1.88	2
12.64	Afzelechin I	$C_{15}H_{14}O_5$	273.0757	2.93	2
13.14	Afzelechin II	$C_{15}H_{14}O_5$	273.0757	2.93	2
15.47	Afzelechin III	$C_{15}H_{14}O_5$	273.0757	2.93	2
	Flavanones				
11.2	Eriodictyol hexoside	$C_{21}H_{22}O_{11}$	449.1078	1.11	2
14.07	Eriodictyol	$C_{15}H_{12}O_6$	287.055	2.79	2
12.18	Naringenin hexoside	$C_{21}H_{22}O_{10}$	433.1129	1.39	2
15.59	Naringenin	$C_{15}H_{12}O_5$	271.0601	3.32	2
	Flavanonols				
11.59	Taxifolin	$C_{15}H_{12}O_7$	303.0499	3.63	2
	Stilbenes				

11.04	Resveratrol hexoside	$C_{20}H_{22}O_8$	389.1231	2.31	2

[a] Annotation from the Summer et al. Compounds identified at MSIMI level 1 means there was a commercially available standard. Compounds identified at MSIMSI level 2 means it was tentativelly identified. RT: retention time.

Table S2. Influence of the cultivar and harvesting season on the galloyl derivatives group (µg/100 g)

	Mono-galloyl-glucose I	Mono-galloyl-glucose II	Galloyl dihexose	Digalloyl hexose I	Digalloyl hexose II	Hydroxy-methoxy phenyl galloyl hexoside I	Hydroxy-methoxy phenyl galloyl hexoside II	Tetra-O-galloyl glucoside I	Tetra-O-galloyl glucoside II	Penta galloyl glucose	Total
**Values[a]**											
Variety											
Aegina	0.32 cd	2.01 ab	0.27 abc	0.18 bc	0.14 ab	0.178 cd	0.49 b	0.134 a	0.73 ab	0.58 ab	5.0 abc
Avdat	0.47 cd	1.90 ab	0.22 abc	0.20 bc	0.14 ab	0.26 4b	0.52 ab	0.124 b	0.64 bc	0.55 bc	5.0 abc
Golden Hills	0.63 cd	1.95 ab	0.30 a	0.15 bc	0.22 ab	0.128 efg	0.15 c	0.099 d	0.31 de	0.23 de	4.2 bcd
Joley	0.20 d	1.51 bc	0.21 bc	0.20 bc	0.14 ab	0.143 def	0.19 c	0.109 c	0.38 d	0.29 d	3.4 d
Kalehghouchi	0.23 cd	1.77 b	0.27 abc	0.13 c	0.11 b	0.094 g	0.10 c	0.097 d	0.23 e	0.16 e	3.2 d
Kastel	1.32 ab	2.46 a	0.26 abc	0.18 bc	0.13 ab	0.099 fg	0.12 c	0.098 d	0.37 d	0.25 de	5.3 ab
Kerman	1.96 a	2.15 ab	0.28 abc	0.32 a	0.26 a	0.152 de	0.24 c	0.099 d	0.43 d	0.29 d	6.2 a
Larnaka	0.88 bc	2.01 ab	0.24 abc	0.22 b	0.15 ab	0.354 a	0.61 ab	0.130 ab	0.7 6ab	0.67 a	6.0 a
Lost Hills	0.35 cd	1.52 bc	0.27 ab	0.16 bc	0.16 ab	0.130 efg	0.18 c	0.104 cd	0.43 d	0.33 d	3.6 cd
Mateur	0.42 cd	2.00 ab	0.27 abc	0.20 bc	0.15 ab	0.177 cd	0.54 ab	0.132 ab	0.79 a	0.63 ab	5.3 ab
Sirora	0.76 bcd	0.95 c	0.19 c	0.21 b	0.13 ab	0.209 c	0.68 a	0.112 c	0.58 c	0.48 c	4.3 bcd
Season											
2019	0.51 b	1.89	0.26	0.18 b	0.15	0.159 b	0.31 b	0.117 a	0.51	0.41	4.5
2020	0.86 a	1.79	0.24	0.21 a	0.16	0.191 a	0.38 a	0.108 b	0.51	0.41	4.9
**Significance[b]**											
Variety (V)	***	***	***	***	*	***	***	***	***	***	***
Season (S)	***	ns	ns	*	ns	***	**	***	ns	ns	ns
V x S	*	ns	ns	***	ns	*	***	**	***	**	*

[a]Average values. [b]Level of significance: *p ≤ 0.05, **p ≤ 0.01, ***p ≤ 0.001, ns: non-significant.
Values with different letters are significantly different as determined through Tukey test.

Table S3. Influence of the cultivar and harvesting season on the hydroxycinnamic acids group (μg/100 g)

	Chlorogenic acid I	Chlorogenic acid II	Ferulic acid derivative	Sinapic acid derivative	p-Coumaric acid	Total
**Values**[a]						
Variety						
Aegina	0.152 abc	0.156 b	< LOQ b	0.035 cd	< LOQ	0.344 b
Avdat	0.155 a	0.155 bc	< LOQ b	0.091 a	< LOQ	0.399 b
Golden Hills	0.147 d	0.154 bcd	< LOQ b	0.063 b	< LOQ	0.365 b
Joley	0.152 abc	0.150 de	< LOQ b	0.030 cd	< LOQ	0.328 b
Kalehghouchi	0.149 bcd	0.153 bcde	< LOQ b	0.006 e	< LOQ	0.308 b
Kastel	0.148 cd	0.149 e	3.065 a	0.040 c	< LOQ	1.870 a
Kerman	0.147 d	0.155 bc	< LOQ b	0.007 e	< LOQ	0.308 b
Larnaka	0.153 ab	0.153 bcde	< LOQ b	0.073 ab	< LOQ	0.379 b
Lost Hills	0.146 d	0.156 b	< LOQ b	0.007 e	< LOQ	0.310 b
Mateur	0.149 bcd	0.161 a	< LOQ b	0.017 de	< LOQ	0.327 b
Sirora	0.146 d	0.151 cde	< LOQ b	0.035 cd	< LOQ	0.332 b
Season						
2019	0.150	0.155 a	3.065 a	0.050 a	< LOQ	0.632 a
2020	0.149	0.153 b	< LOQ b	0.024 b	< LOQ	0.325 b
**Significance**[b]						
Variety (V)	***	***	***	***	ns	***
Season (S)	ns	*	***	***	ns	***
V x S	*	ns	***	***	ns	***

[a]Average values. [b]Level of significance: *p ≤ 0.05, **p ≤ 0.01, ***p ≤ 0.001, ns: non-significant.
Values with different letters are significantly different as determined through Tukey test.

Table S4. Influence of the cultivar and harvesting season on the flavones group (µg/100 g)

	Luteolin hexoside	Luteolin	Apigenin hexoside	Apigenin	Total
**Values[a]**					
Variety					
Aegina	3.28 abc	0.024 e	< LOQ	0.012 ab	3.31 ab
Avdat	4.09 ab	0.016 e	< LOQ	0.008 c	4.12 a
Golden Hills	0.32 g	0.109 cde	< LOQ	0.011 abc	0.44 f
Joley	1.97 def	0.066 de	< LOQ	0.008 bc	2.04 cde
Kalehghouchi	2.01 def	0.294 b	< LOQ	0.009 abc	2.32 bcde
Kastel	1.76 ef	0.191 bc	< LOQ	0.009 abc	1.96 de
Kerman	1.87 ef	0.144 cd	< LOQ	0.011 abc	2.03 cde
Larnaka	4.33 a	0.017 e	< LOQ	0.009 abc	4.36 a
Lost Hills	0.87 fg	0.519 a	< LOQ	0.011 abc	1.40 ef
Mateur	2.87 cde	0.032 de	< LOQ	0.012 a	2.91 bcd
Sirora	3.07 bcd	0.102 cde	< LOQ	0.008 bc	3.18 abc
Season	3.28 abc	0.024 e	< LOQ	0.012 ab	3.31 ab
2019	1.89 b	0.122 b	< LOQ	0.011 a	2.03 b
2020	2.91 a	0.153 a	< LOQ	0.009 b	3.08 a
**Significance[b]**					
Variety (V)	***	***	ns	***	***
Season (S)	***	*	ns	*	***
V x S	**	***	ns	ns	**

[a]Average values. [b]Level of significance: *p ≤ 0.05, **p ≤ 0.01, ***p ≤ 0.001, ns: non-significant.
Values with different letters are significantly different as determined through Tukey test.

Table S5. Influence of the cultivar and harvesting season on the flavonols group (µg/100 g)

	Rutin	Myricetin hexoside	Myricetin	Quercetin hexoside I	Quercetin hexoside II	Quercetin galloyl glucoside I	Quercetin galloyl glucoside II	Quercetin	Isorhamnetin	Total
**Values**[a]										
Variety										
Aegina	0.37 bc	0.69 a	0.038 ab	0.226 a	2.71 a	0.071 d	0.019 ab	0.17 b	0.012	4.31 a
Avdat	0.31 cde	0.60 ab	0.025 bcd	0.180 abc	2.49 ab	0.195 b	0.005 cd	0.13 b	0.010	3.95 abcd
Golden Hills	0.23 f	0.14 d	0.020 cd	0.089 de	1.21 d	0.003 e	0.005 cd	0.19 b	0.013	1.89 f
Joley	0.61 a	0.16 d	0.012 d	0.126 cd	2.00 bc	0.091 cd	< LOQ	0.14 b	0.012	3.14 de
Kalehghouchi	0.27 def	0.36 c	0.026 bcd	0.043 e	1.49 cd	0.138 c	nd	0.15 b	0.010	2.49 ef
Kastel	0.22 f	0.41 c	0.027 bcd	0.151 bcd	1.52 cd	0.011 e	< LOQ	0.13 b	0.012	2.49 ef
Kerman	0.26 ef	0.45 bc	0.021 cd	0.144 bcd	2.35 ab	0.043 de	< LOQ	0.14 b	0.015	3.42 bcd
Larnaka	0.32 bcd	0.63 a	0.031 bc	0.187 abc	2.57 ab	0.288 a	0.015 ab	0.16 b	0.012	4.22 abc
Lost Hills	0.25 ef	0.12 d	0.019 cd	0.161 abcd	2.29 ab	0.000 e	0.005 cd	0.28 a	0.017	3.15 de
Mateur	0.38 b	0.76 a	0.052 a	0.201 ab	2.59 ab	0.075 d	0.023 a	0.19 ab	0.018	4.30 ab
Sirora	0.33 bcd	0.37 c	0.018 cd	0.126 cd	2.35 ab	0.017 e	0.011 bc	0.15 b	0.013	3.38 cd
	0.37 bc	0.69 a	0.038 ab	0.226 a	2.71 a	0.071 d	0.019 ab	0.17 b	0.012	4.31 a
Season										
2019	0.35 a	0.37 b	0.017 b	0.142	2.05 b	0.072 b	0.003 b	0.15 b	0.010 b	3.16 b
2020	0.30 b	0.49 a	0.035 a	0.155	2.24 a	0.098 a	0.012 a	0.18 a	0.016 a	3.52 a
**Significance**[b]	0.35 a	0.37 b	0.017 b	0.142	2.05 b	0.072 b	0.003 b	0.15 b	0.010 b	3.16 b
Variety (V)	***	***	***	***	***	***	***	***	ns	***
Season (S)	***	***	***	ns	*	***	***	**	***	**
V x S	***	***	***	*	*	ns	***	ns	*	**

[a]Average values. [b]Level of significance: *p ≤ 0.05, **p ≤ 0.01, ***p ≤ 0.001, ns: non-significant.
Values with different letters are significantly different as determined through Tukey test.

Table S6. Influence of the cultivar and harvesting season on the flavanones group (µg/100 g)

	**Eriodictyol hexoside**	**Eriodictyol**	**Naringenin hexoside**	**Naringenin**	**Total**
**Values**[a]					
Variety					
Aegina	6.7 3a	1.04 a	0.286 a	0.043 bcd	8.10 a
Avdat	5.04 b	0.89 ab	0.273 a	0.049 b	6.24 b
Golden Hills	0.90 c	0.32 d	0.063 b	0.049 b	1.34 cd
Joley	4.05 b	0.85 abc	0.256 a	0.069 a	5.22 b
Kalehghouchi	0.91 c	0.58 bcd	0.054 b	0.052 b	1.60 cd
Kastel	0.77 c	0.21 d	0.037 b	0.026 e	1.05 d
Kerman	1.32 c	0.32 d	0.059 b	0.022 e	1.72 cd
Larnaka	4.88 b	0.86 abc	0.253 a	0.046 bcd	6.04 b
Lost Hills	1.13 c	0.48 cd	0.066 b	0.034 cde	1.70 cd
Mateur	6.92 a	1.03 a	0.311 a	0.047 bc	8.31 a
Sirora	1.76 c	0.55 bcd	0.079 b	0.033 de	2.42 c
Season					
2019	2.83 b	0.71 a	0.144 b	0.042	3.73 b
2020	3.42 a	0.58 b	0.172 a	0.044	4.22 a
**Significance**[b]					
Variety (V)	***	***	***	***	***
Season (S)	***	*	**	ns	**
V x S	***	ns	***	*	***

[a]Average values. [b]Level of significance: *p ≤ 0.05, **p ≤ 0.01, ***p ≤ 0.001, ns: non-significant.
Values with different letters are significantly different as determined through Tukey test.

Table S7. Influence of the cultivar and harvesting season on the flavanonols group (µg/100 g)

	**Taxifolin**
**Values**[a]	
Variety	
Aegina	1.15
Avdat	0.90
Golden Hills	0.44
Joley	0.68
Kalehghouchi	1.23
Kastel	0.46
Kerman	0.83
Larnaka	0.76
Lost Hills	0.78
Mateur	0.95
Sirora	0.88
Season	
2019	1.11 a
2020	0.54 b
**Significance**[b]	
Variety (V)	ns
Season (S)	***
V x S	ns

[a]Average values. [b]Level of significance: *p ≤ 0.05, **p ≤ 0.01, ***p ≤ 0.001, ns: non-significant.
Values with different letters are significantly different as determined through Tukey test.

Table S8. Influence of the cultivar and harvesting season on the stilbenes group (µg/100 g)

	**Resveratrol hexoside**
**Values[a]**	
Variety	
Aegina	4.20 b
Avdat	5.22 a
Golden Hills	1.40 d
Joley	2.12 cd
Kalehghouchi	1.66 cd
Kastel	2.00 cd
Kerman	2.47 c
Larnaka	4.92 ab
Lost Hills	1.88 cd
Mateur	4.08 b
Sirora	2.41 c
Season	
2019	2.81 b
2020	3.08 a
**Significance[b]**	
Variety (V)	***
Season (S)	*
V x S	*

[a]Average values. [b]Level of significance: *p ≤ 0.05, **p ≤ 0.01, ***p ≤ 0.001, ns: non-significant.
Values with different letters are significantly different as determined through Tukey test.
